# Intraspinal migration of a Kirschner wire as a late complication of acromioclavicular joint repair: a case report

**DOI:** 10.1186/s13256-016-0844-4

**Published:** 2016-03-24

**Authors:** Bartosz Mankowski, Tadeusz Polchlopek, Marcin Strojny, Pawel Grala, Krzysztof Slowinski

**Affiliations:** Department of Trauma, Burns and Plastic Surgery, Poznan University of Medical Sciences, Poznań, Poland

**Keywords:** Implant migration, K-wire migration, Penetrating neck trauma, Surgical management of neck trauma, Vertebral artery injury

## Abstract

**Background:**

Penetrating neck trauma involving foreign bodies is a rare event in European countries. Due to its relatively high mortality rate, the correct management strategy must be initiated from the beginning to prevent fatal complications. In the medical literature, there are only a few cases describing foreign bodies penetrating the cervical spine. Because of its rareness, many trauma centers lack the proper routine to adequately manage such injuries.

**Case presentation:**

This case report describes a 34-year-old white man of Central European descent with Kirschner wire migration and perforation of his vertebral foramen. He underwent acromioclavicular joint repair surgery 7 years ago, presented with a painful sensation around the area of his left clavicle and left side of his neck after a motorcycle accident. No neurological deficit was detected.

**Conclusions:**

In such cases, a thorough radiological evaluation of the spinal cord and the surrounding vasculature is mandatory for a complete understanding of the extent of the injury and determining the proper surgical management. In cases of vertebral artery trauma both an endovascular and an open approach can be contemplated.

## Background

Penetrating neck injuries are uncommon in European countries. Due to the lack of cases, surgeons have insufficient experience to properly deal with such problems. The complexity of neck anatomy makes procedures in this region very challenging and can easily lead to complications such as airway compromise, severe hemorrhage, and peripheral or cranial nerve damage [1–3]. Injuries caused by foreign bodies in the neck are uncommon as well, they usually occur as a result of ingested objects penetrating the cervical area. In the medical literature, there are only a few cases describing foreign bodies penetrating the cervical spine [4, 5]. In addition, injuries to the vertebral artery caused by a foreign body penetrating the cervical spine are extremely rare. Vertebral artery injuries are scarce even in specialized trauma centers [6]. The incidence of vertebral artery injury varies from 0.5 to 2 % in all trauma cases [7–9] and represents less than 1 % of all vascular injuries [10]. However, the mortality rate of traumatic vertebral artery injury is reported to be approximately 8 % [11–13]. Symptoms of vertebral artery injury are associated with ischemia of the cerebellum, brain stem, and the primary visual cortex [7].

## Case presentation

**Fig. 1:**
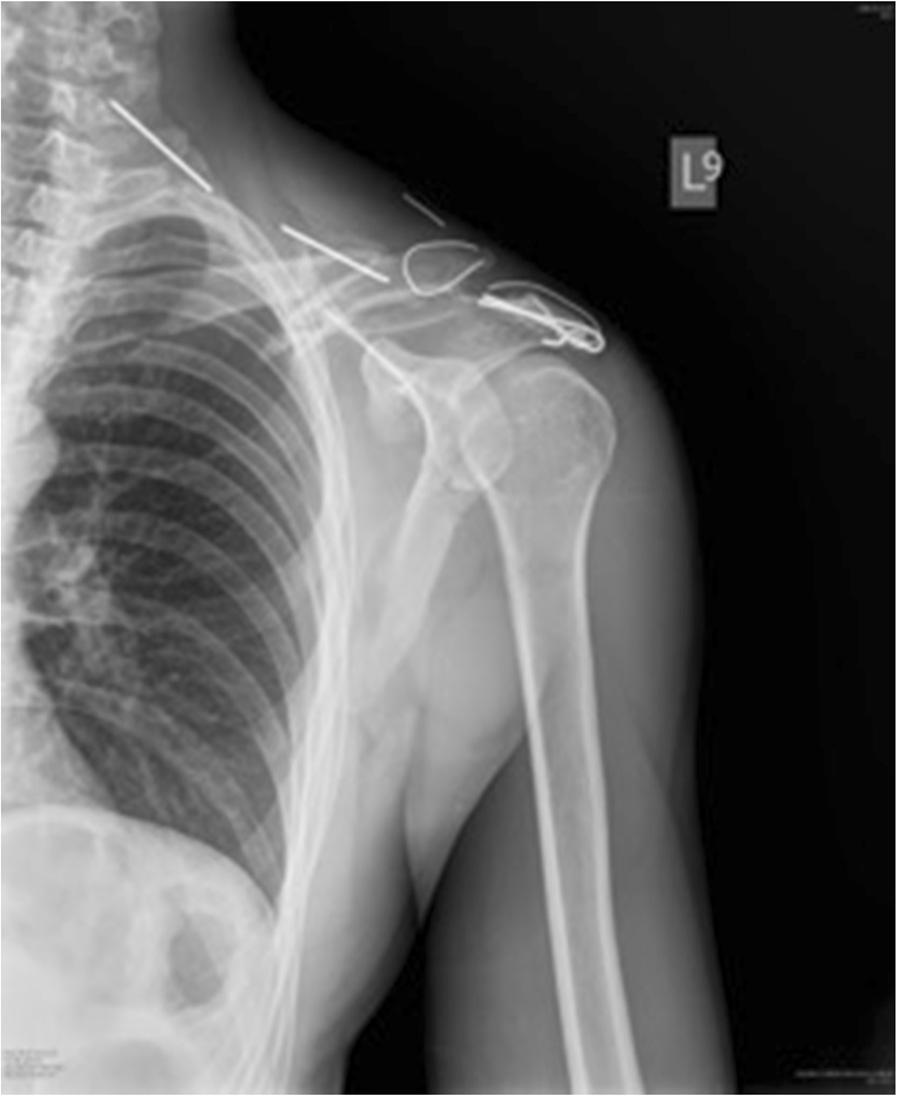
Shoulder radiogram shows migration of Kirschner wires toward cervical spine

**Fig. 2:**
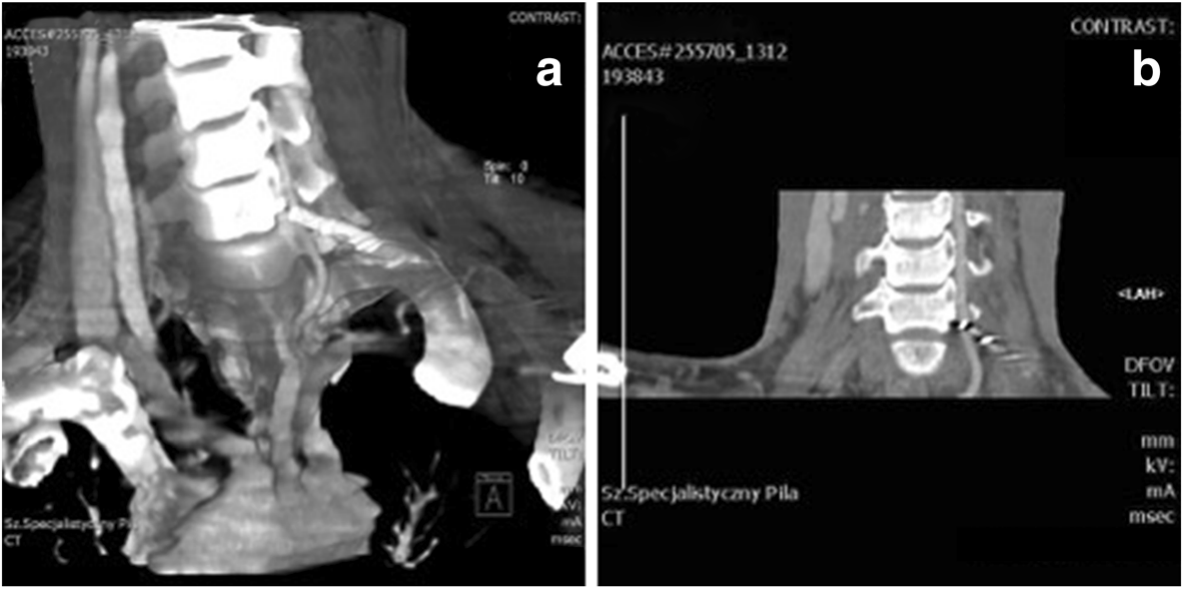
**a** Angio-computed tomography of cervical spine; Kirschner wire penetration of C6 vertebra. **b** Angio-computed tomography of cervical spine; direct contact of Kirschner wire with vertebral artery without breaching the lumen

**Fig. 3:**
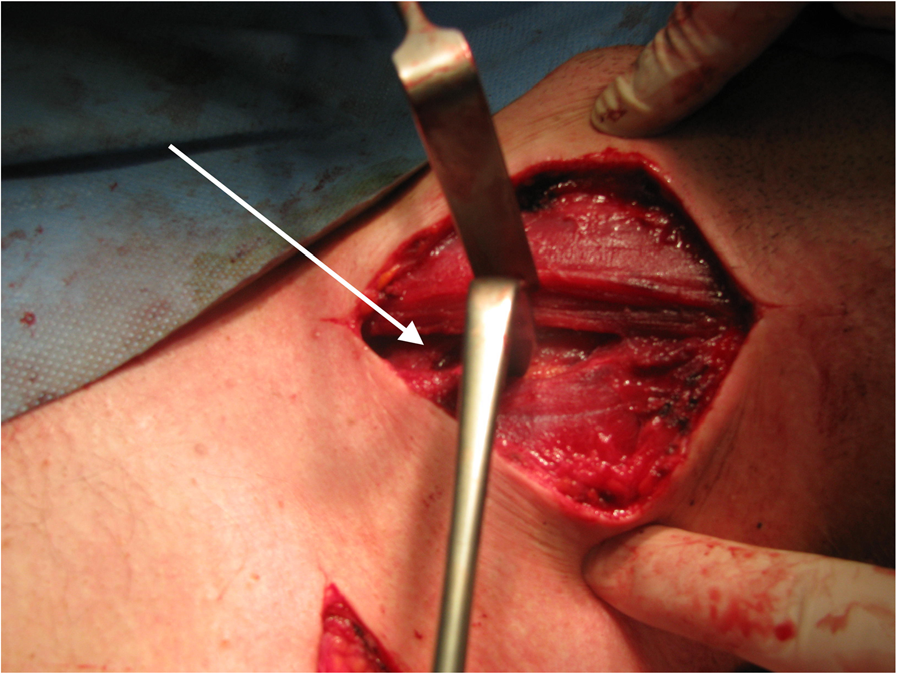
Open surgical management. Incision made parallel to the lateral wall of sternocleidomastoid muscle. *Arrow* indicates the migrating Kirschner wire

**Fig. 4:**
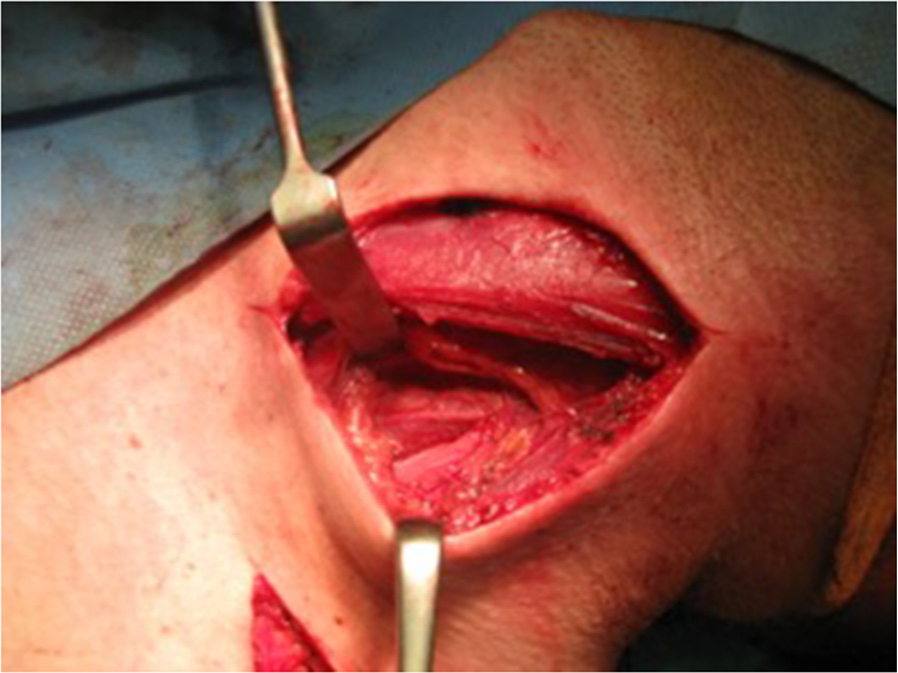
Visualization of scalene muscles

A 34-year-old white man of Central European descent was admitted to our department after falling off a motorcycle onto his left shoulder. He had undergone an acromioclavicular joint repair procedure with the use of two Kirschner wires (K-wires) and a tension band 7 years ago. The implants were never removed. His chief complaint was of painful sensation around the area of his left clavicle and left side of his neck. No neurological deficit was detected. An X-ray performed at our department showed two broken K-wires and a tension band. One of the wires penetrated the suprascapular soft tissues; the other had migrated toward his cervical spine (Fig. 1). A computed tomography angiography revealed that the K-wire penetrated his C6 vertebra (Fig. 2a) and was in direct contact with his vertebral artery without breaching the lumen (Fig. 2b). Furthermore, a dislocated left clavicle shaft fracture was detected without injury to the underlying vascular and neural structures. After proper diagnosis and preparation, he was scheduled for an elective surgery. During surgery, the position of the K-wire was located by anterior-posterior fluoroscopy. A skin incision was made parallel to the lateral wall of his sternocleidomastoid muscle (SCM) as seen in Fig. 3. His platysma muscle was then incised and the SCM, with the cervical vessels, was retracted medially. Further exploration of the lateral cervical triangle was carried out by careful blunt and sharp dissection to reach components of the brachial plexus and to visualize the scalene muscles (Fig. 4).

**Fig. 5:**
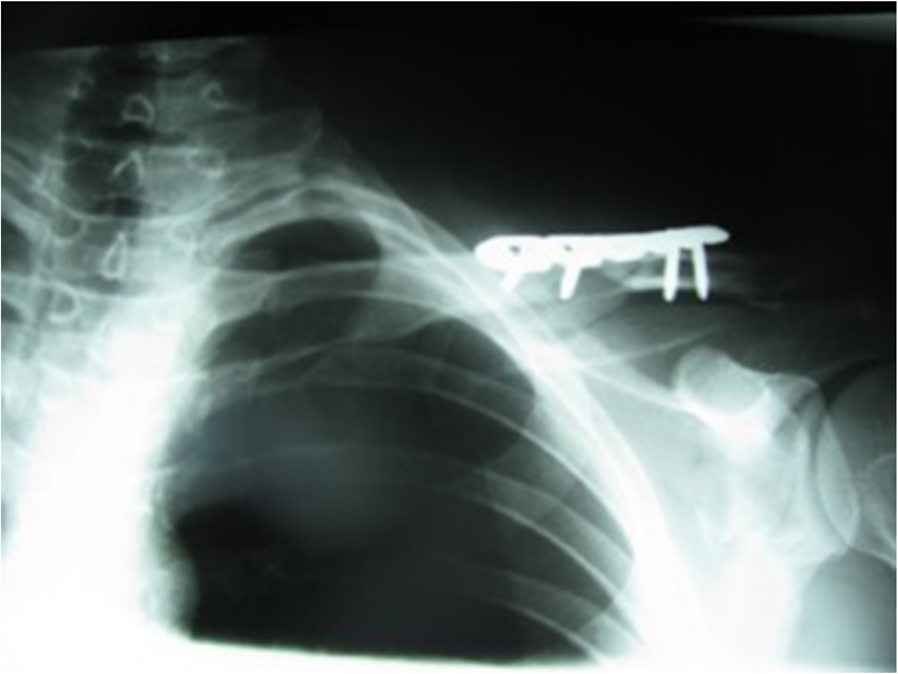
Open reduction and internal fixation of fractured clavicle using locking compression plate

At this time the K-wire was not palpable and further dissection between the scalene muscles was necessary. When the K-wire became visible, the free end was dissected from the surrounding tissues and removed. No injury to his brachial plexus was detected and no bleeding was noted. The remaining wires were removed from his clavicle and supraclavicular area without complications through a second incision performed alongside the clavicle. Fluoroscopy was performed to determine the location of the remaining implants and to make sure that no other metal components were overlooked. Through the same incision the fractured clavicle was reduced and stabilized using a locking compression plate (LCP) system as seen in Fig. 5. During the post-surgical evaluation a paresthesia was noted on his C8 dermatome radiating to his fourth and fifth finger. However, this resolved spontaneously within 3 days. He was discharged on the seventh day with no neurological deficits. The angiography unit was informed about the operation and possible intraoperative vertebral artery injury, in the event endovascular intervention would be needed.

## Discussion

Penetrating neck injuries are stressful events for surgeons. The risk of iatrogenic damage to surrounding structures is high and makes flawless knowledge of the neck anatomy mandatory. K-wire migration into the cervical spine after fixation of a fractured clavicle is rare. Apart from injury to the vertebral artery, it may cause damage to the nerve roots, dura mater, and spinal cord. When such material penetrates the vertebral canal, a wide laminectomy is required to expose both ends of the K-wire, followed by dura mater repair and hemostasis [4, 5]. In the presented case there was a risk of causing injury to the vertebral artery while removing the K-wire. Mwipatayi *et al*. never attempted to repair the vertebral artery in cases with such an injury. In all cases the vessel was ligated, clipped or hemostasis was attained using bone wax [6].

In general, the vertebral artery can be divided in four anatomical segments: V1 to V4 [14, 15]. Care must be taken to account for anatomical variations of these segments to avoid serious complications by iatrogenic injury [16]. The first part of vertebral artery (V1) originates from the subclavian artery and ends by entering the transverse foramen. In 90 % of cases, its entrance is at the level of C6, but it can be as high as C3 [16]. The artery is relatively unprotected during this path and its injury would require wide surgical exposure and ligation of the vessel. The V2 segment travels through the C6 to C2 transverse processes and merges into the V3 from the C2 vertebra to its entry point through the dura mater [14]. Bleeding, due to arterial injury, in these segments should be stopped using bone wax. However, if performed carelessly, this may harm the cervical nerve root. The last part of the vertebral artery (V4) has an entirely intracranial course, merging with the basilar artery. Occlusion of the vertebral artery at this point may cause cerebellar ischemia. Other complications, due to injury to the vertebral artery, are massive neck hematomas, pseudoaneurysms, dissections or arteriovenous fistulas [6]. The mortality rate associated with vertebral artery injury is estimated to be 6.9 % [6]. Apart from severe and uncontrollable bleeding, most vertebral artery injuries are asymptomatic. Reid and Weigelt suggested that neurological deficits accompanying vertebral artery injury are caused by direct physical damage to the spinal cord and cervical roots rather than ischemic changes within these structures [17]. An open neck exploration procedure is the preferred method for acute and unstable cases with an uncontrolled hemorrhage and a growing hematoma in the cervical region [6]. Stable patients, that is, vertebral dissections, can be treated by endovascular techniques, which are the recommended procedures compared to an open surgical intervention. These techniques allow vascular repair using a minimal invasive approach and have proven their value in earlier studies [18]. Blunt vertebral artery injuries can be successfully managed as well using endovascular techniques including stenting, occlusion or pseudoaneurysm coil occlusion [6, 7, 18, 19]. Lesions that do not qualify for endovascular treatment are those that are within 2 cm from the origin of the vertebral artery or in the V4 segment close to the posterior inferior cerebral artery (PICA) [6]. Herrera *et al*. presented the endovascular treatment of 18 patients with penetrating injury of the vertebral artery. In all cases, sacrifice of the artery was a necessity due to severe hemorrhage. Occlusion was carried out if patency of the PICA was visualized. The authors did not observe any neurological complications; the unaffected vertebral artery seemed to sufficiently supply the contralateral circulation [19].

The presented case can be subdivided into two main categories of surgical management: open surgery or endovascular procedures. The open approach was necessary to safely remove the foreign body and to prevent vertebral artery injury. If, during surgery, a hemorrhage had occurred, direct pressure could have been applied to achieve hemostasis. In case of an uncontrollable bleeding, the patient would have been moved to the angiography unit for endovascular occlusion of the vessel.

## Conclusions

Vertebral artery injuries are uncommon and rarely caused by penetrating foreign bodies. The possibility of injury to the vertebral artery must be evaluated which makes accurate radiological assessment mandatory. An open surgical approach is still the recommended management procedure for the acute setting. Endovascular techniques have proven their value in penetrating vertebral artery injuries and are capable of achieving hemostasis in severe hemorrhages.

## Patient’s perspective

I experienced no problems with my shoulder joint following initial surgery to my acromioclavicular joint 7 years ago. After my motorcycle accident, I was taken by the ambulance to an emergency department where they said I needed surgery. The surgeon explained in detail the possible risks and complications that could occur during surgery. Nonetheless, I agreed to have the surgery done at a trauma centre in Poznan, Poland. Surgery went without complications and I was discharged home 3 days after surgery. I have no medical knowledge and I write the above only to assist in this case report.

## Consent

Written informed consent was obtained from our patient for the publication of this case report and any accompanying images. A copy of the written consent is available for review by the Editor-in-Chief of this journal.
